# Osteosarcoma enters a post genomic era with *in silico* opportunities: Generation of the High Dimensional Database for facilitating sarcoma biology research: A report from the Children's Oncology Group and the QuadW Foundation

**DOI:** 10.1371/journal.pone.0181204

**Published:** 2017-07-21

**Authors:** Jason Glover, Tsz-Kwong Man, Donald A. Barkauskas, David Hall, Tanya Tello, Mary Beth Sullivan, Richard Gorlick, Katherine Janeway, Holcombe Grier, Ching Lau, Jeffrey A. Toretsky, Scott C. Borinstein, Chand Khanna, Timothy M. Fan, for the COG Osteosarcoma Biology Group

**Affiliations:** 1 Children's Cancer and Blood Disorders Program, Randall Children's Hospital, Portland, Oregon, United States of America; 2 Department of Pediatrics, Baylor College of Medicine, and Texas Children’s Cancer Center, Houston, Texas, United States of America; 3 Department of Preventive Medicine, Keck School of Medicine of the University of Southern California, Los Angeles, California, United States of America; 4 QuadW-COG Childhood Sarcoma Biostatistics and Annotation Office, Children’s Oncology Group, Monrovia, California, United States of America; 5 Department of Pediatrics, University of Texas, MD Anderson Cancer Center, Houston, Texas, United States of America; 6 Department of Pediatrics, Harvard Medical School, Dana-Farber/Boston Children’s Cancer and Blood Disorders Center, Boston, Massachusetts, United States of America; 7 Center for Cancer and Blood Disorders, Connecticut Children's Medical Center, Hartford, Connecticut, United States of America; 8 Department of Oncology, Georgetown University Medical Center, Washington DC, United States of America; 9 Department of Pediatrics, Vanderbilt University Medical Center, Nashville, Tennessee, United States of America; 10 Ethos Discovery in Washington DC and Ethos Veterinary Health, Woburn, Massachusetts, United States of America; 11 Department of Veterinary Clinical Medicine, University of Illinois, Urbana, Illinois, United States of America; Universite de Nantes, FRANCE

## Abstract

The prospective banking of osteosarcoma tissue samples to promote research endeavors has been realized through the establishment of a nationally centralized biospecimen repository, the Children’s Oncology Group (COG) biospecimen bank located at the Biopathology Center (BPC)/Nationwide Children’s Hospital in Columbus, Ohio. Although the physical inventory of osteosarcoma biospecimens is substantive (>15,000 sample specimens), the nature of these resources remains exhaustible. Despite judicious allocation of these high-value biospecimens for conducting sarcoma-related research, a deeper understanding of osteosarcoma biology, in particular metastases, remains unrealized. In addition the identification and development of novel diagnostics and effective therapeutics remain elusive. The QuadW-COG Childhood Sarcoma Biostatistics and Annotation Office (CSBAO) has developed the High Dimensional Data (HDD) platform to complement the existing physical inventory and to promote *in silico* hypothesis testing in sarcoma biology. The HDD is a relational biologic database derived from matched osteosarcoma biospecimens in which diverse experimental readouts have been generated and digitally deposited. As proof-of-concept, we demonstrate that the HDD platform can be utilized to address previously unrealized biologic questions though the systematic juxtaposition of diverse datasets derived from shared biospecimens. The continued population of the HDD platform with high-value, high-throughput and mineable datasets allows a shared and reusable resource for researchers, both experimentalists and bioinformatics investigators, to propose and answer questions *in silico* that advance our understanding of osteosarcoma biology.

## Introduction

Osteosarcoma is a cancer of the bone that is most common in the pediatric and young adult population [1]. The therapeutic management of osteosarcoma has not substantively changed since the introduction of systemic chemotherapy in the 1980’s, and consequently survival rates for osteosarcoma have remained largely static for the past 3 decades. In a minority, yet substantive percent of patients (~30%), cure remains unachievable given the development of recurrent or metastatic disease [1]. For these high risk patients, there is strong clinical and scientific impetus for increasing the fundamental understanding of osteosarcoma biology, in particular the cellular mechanisms of metastases. The ultimate goal shared by clinicians and scientists would be the identification and development of rational treatment options for osteosarcoma patients at high risk for disease relapse or progression.

The creation of a large physical inventory of biospecimens remains critically important for driving discoveries in diverse tumor types including osteosarcoma. However, a complementary and alternative mechanism for sarcoma biology discovery can be through the creation of a reusable biological database derived from the findings of past, ongoing, and future research investigations. Expected outcomes of such a shared resource would be multifold, including timely data sharing by investigative groups for expediting new and global discoveries, minimizing redundant or repetitive investigations which carry the risk for depleting exhaustible banked biospecimens, and encouraging the practice of *in silico* hypothesis testing by both conventional experimentalists and bioinformatics investigators that requires minimal upfront resource allocations and can accelerate the development of more focused research efforts.

The Children’s Oncology Group and the QuadW Foundation have recognized the need to quickly develop biology projects and conduct research related to childhood sarcomas in a cost effective manner [2]. Banked biospecimens are a finite resource and require ongoing infrastructural costs to maintain, and hence a strong motivation exists to establish a digital biologic database which allows for facile and recurring exploratory investigations pertaining to sarcoma biology. Another recognized benefit of the HDD is enabling access to a broader group of researchers with expertise in bioinformatics and data mining, who have divergent scientific perspectives capable of asking innovative questions derived from the inclusion of multiple “omics” and clinically annotated data, which otherwise would remain unrealized in the existing osteosarcoma research community. To address the potential needs of a wider clinical and research community, a scalable data management platform has been developed from voluntarily shared data derived from diverse and historical investigative studies conducted in osteosarcoma biology. These investigative studies utilized osteosarcoma biospecimens obtained through the Children’s Oncology Group protocols. Here within, we describe the processes and datasets used to establish the HDD platform and demonstrate proof-of-concept *in silico* hypothesis testing by combining results from 2 disparate investigations examining gene microarray analysis and circulating concentrations of insulin-like growth factor binding protein 2 (IGFBP2).

## Materials and methods

### Identification and transfer of datasets

Biospecimen requests from sarcoma researchers spanning 2004–2014, which satisfied scientific criteria to justify dispersion of biologic samples from the Children’s Oncology Group (COG) biospecimen bank located at the Biopathology Center (BPC)/Nationwide Children’s Hospital, were identified and used to develop a preliminary project list (tier 1). All biospecimen analyses have been conducted in accordance to IRB approved guidelines and specimens were obtained with written informed consent for enrollment on COG biology studies. An additional filter (tier 2) was applied to the preliminary project list, being biospecimen requests whereby infrastructural support was provided by the QuadW Childhood Sarcoma Biostatistics and Annotation Office (CSBAO) to research investigators requiring data analysis and/or data transfer support, and a final project list was generated. Individual research investigators identified on the final project list were contacted by Osteosarcoma Biology committee leadership with the request for voluntary data sharing and digital data importation into the HDD platform. Programming of the HDD platform is written using Ruby on Rails, with the creation of both “backroom” and “front room” components using ASP.NET with Microsoft’s MVC framework and SQL Server and SQL Server Reporting Services and Integrated Services (SSRS and SSIS). The file flow components utilize Apache Camel for creating data integration patterns and routes which allow for data sets to be recognized and uploaded upon principal investigator transfer. Both raw and normalized datasets were imported depending upon research investigator preference, with typical shared data being provided by investigators in the form of Excel files which allow for facile data integration. In addition, other file types including CEL files and other data formats can be uploaded into the HDD system using Nationwide Children’s Hospital secure FTP sites for small datasets or Globus for datasets greater than 3 GB. Population and maintenance of digital information shared voluntarily by research investigators was performed by the informatics team at BPC (Heather Day).

### Proof-of-concept in silico hypothesis testing

The number of patient derived samples shared between 2 (pairs) or among 3–5 (trios, quartets, and quintets) investigations were identified from the final project list. Proof-of-concept *in silico* hypothesis testing was performed using biological datasets from overlapping patient-specific biospecimens (pairs; n = 46) derived from the Strategic Partnering to Evaluate Cancer Signatures (SPECS) and IGFBP2 projects. Given the distinct role that IGFBP2 plays in cancer cell metabolism [3, 4], the primary hypothesis to be tested was that 1) circulating IGFBP2 concentrations would be associated with distinct primary tumor gene microarray signatures, and secondarily that 2) the principal source of circulating IGFBP2 was derived from the primary tumor.

The RNA expression profiling results were obtained from the osteosarcoma project of the NCI-SPECS program. In total, 46 RNA expression profiles of osteosarcoma analyzed by Affymetrix U133 plus 2 microarrays were matched to the blood samples that have measured circulating IGFBP2 results [5]. The raw intensities of the probesets were quantile normalization and log-transformed. The probesets with variation less than 75 percentile or missing values exceeding 50% were removed, which resulted in 11,445 probesets for the subsequent analysis. To maximize statistical power based upon the limited number of overlapping samples, some comparisons were based upon grouping of data as either above/below median, while other analysis utilized data expressed as quartiles. To compare the groups with high and low circulating IGFBP2 levels using the median circulating IGFBP2 level as a cutoff, two-sample t-tests were performed with gender as a blocking factor. The confidence level of false discovery rate assessment and the maximum allowed proportion of false-positive genes for identifying differentially expressed genes were set at 80% and 0.1, respectively. In addition, the expression levels of the matched tumors were plotted against the quartiles of the circulating IGFBP2 levels to illustrate the correlation of the IGFBP2 levels between tumor and circulation. Hierarchical clustering with average linkage and correlation was performed to test if the expression profiles of the osteosarcoma cases were clustered separately with respect to the circulating IGFBP2 levels. The analyses were performed using BRB-ArrayTools (V4.5.1) [6].

## Results

### Dataset identification

**Fig 1:**
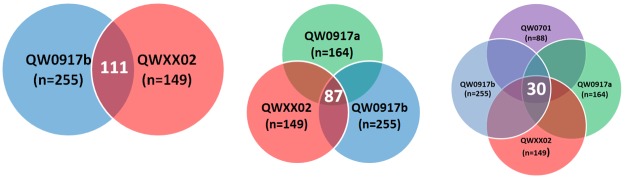
Representative Venn diagrams of shared and overlapping biospecimens used to derive unique assay data (pairs, trios, or quartets) from divergent investigations.

Ten discrete datasets were identified which fulfilled the 2-tier criteria to generate a list of osteosarcoma biology projects where biospecimens were dispersed to research investigators and required QuadW CSBAO infrastructural support (Table 1). Biologic data derived from these identified projects utilized diverse methodologies including histopathology, immunohistochemistry, enzyme linked immunoassays, proteomics, gene microarrays, polymerase chain reaction, fluorescent in situ hybridization, and multiple genome sequencing techniques. The most mineable and potentially highest-value datasets for future *in silico* hypothesis testing included results from SPECS (gene microarray) and the NCI’s Therapeutically Applicable Research to Generate Effective Treatments (TARGET) program that includes genome-wide analysis through multiple platforms. Excluding primary tumor necrosis grading of 974 patient-specific biospecimens, the median number of biospecimens whereby unique bioassays were derived was 148, with a range of 45–255 unique biospecimens (Table 1). The extent and degree of overlapping biospecimens in excess of 10 samples shared between 2 (pairs) or 3–5 (trios, quartets, quintets) independent investigations are summarized in Table 2, with representative schematics underscoring potential biospecimen overlap between and among different data sets (Fig 1).

**Table 1:** Imported digital data within High Dimensional Database platform available for *in silico* research.

Study Identifier	Investigator	N = Samples	Readout	Methodology	Reference
**Therapeutic**	COG	974	Necrosis	Histopathology	
**QW0701**	Koshkina	88	Fas	SNPs	[7]
**QW0801**	Ebb	96	ErbB-2	IHC	[8]
**QW0909**	Lau	143	TARGET	Sequencing	
**QW0917a**	Borinstein	164	IGF-1 axis	ELISA	[5]
**QW0917b**	Borinstein	255	IGFBP-2	ELISA	[9]
**QWXX02**	Gorlick	149	ErbB-2	IHC	[10]
**QWXX05**	Lau	229	SPECS	cDNA array	
**QWXX08**	Ragg	148	Biomarker discovery	Proteomics	
**QWXX1**	Squire	45	CIN	FISH	
**QWXX01**	Dome	123	TERT	PCR	

SNPs- single nucleotide polymorphism; IHC- immunohistochemistry; ELISA- enzyme linked immunosorbent assay; CIN- chromosomal instability; FISH- fluorescent in situ hybridization; TERT- telomerase reverse transcriptase; PCR- polymerase chain reaction; TARGET- therapeutically applicable research to generate effective treatments; SPECS- strategic partnering to evaluate cancer signatures

**Table 2:** Number of common biospecimens (≥ 10) used to derive unique assay data.

Overlapping Biospecimens	Pairs	Trios	Quartets	Quintets
**Maximum**	149	104	62	28
**Minimum**	12	10	10	10
**Median**	33	15.5	14.5	14

### Proof-of-concept testing

**Fig 2:**
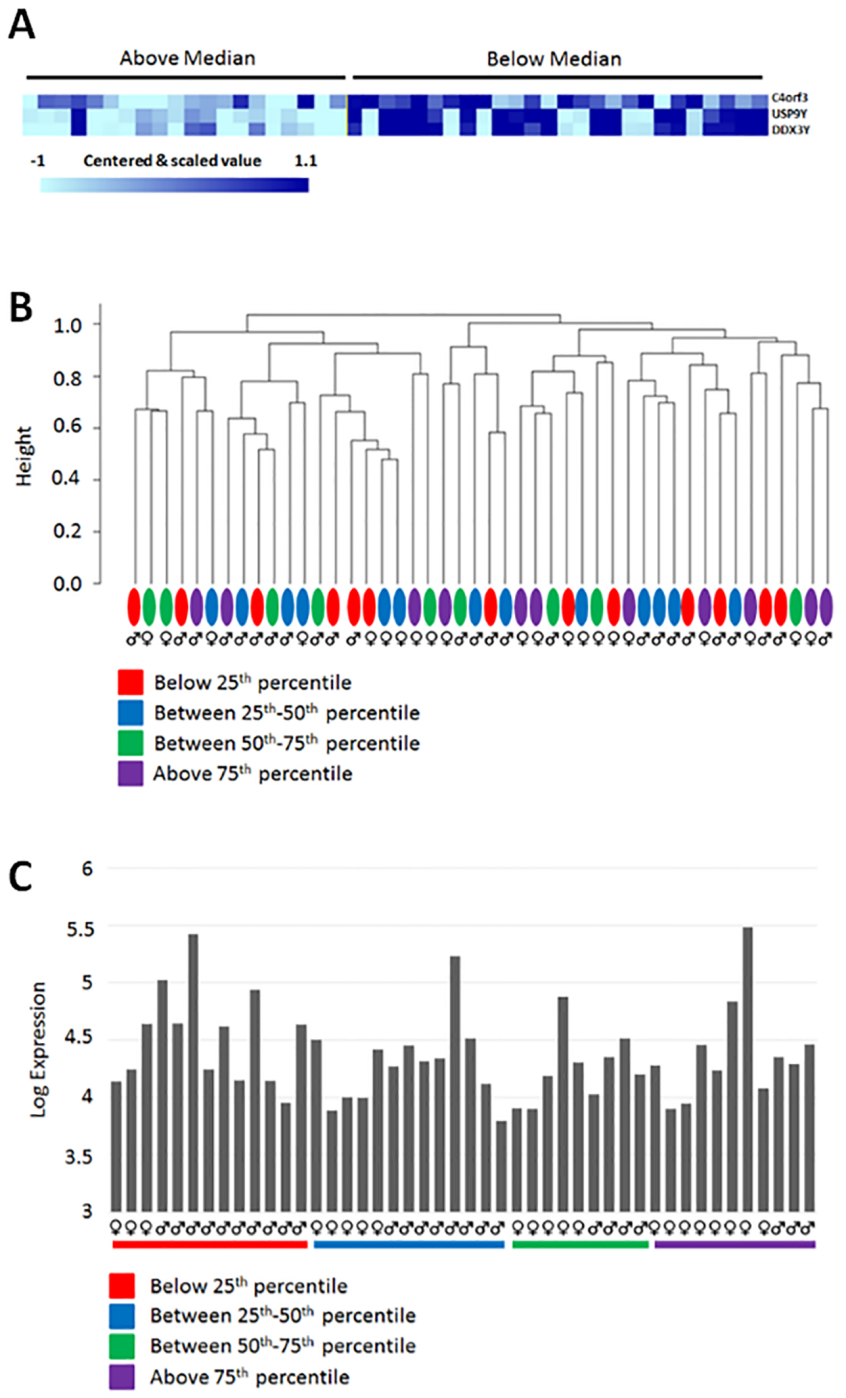
(A) Heat map showing the RNA expression of 3 genes C4orf3, USP9Y, and DDX3Y inversely correlated with circulating concentrations of IGFBP2. (B) Unsupervised hierarchal clustering analysis of gene expression profiles of the primary tumors based upon quartile circulating IGFBP2 concentrations. (C) The plot of primary tumor IGFBP2 mRNA expressions against circulating IGFBP2 concentrations of the matched samples.

To test that ability to ask *in silico* questions using HDD as a biologic relational database, 46 overlapping patient-specific biospecimens used for gene microarray analysis (SPECS) and circulating concentrations of IGFBP2 were evaluated for a differential expression analysis. Circulating concentrations of IGFBP2 were categorized as either median (high/low) or quartiles values (< = 25%, 26–50%, 51–75%, > = 75%). When IGFBP2 concentrations were categorized above or below the median, gender appeared to act as a segregating factor with 13 females in the high expression group (n = 20) and only 8 in the low expression group (n = 26), being 65% and 31%, respectively (*p* = 0.04). With respect to circulating IGFBP2, these findings suggest that more females are in the high expression group than in the low expression group, and are consistent with historical studies in which insulin-like growth factors and/or IGFBP concentrations have been linked to gender [11]. To correct for the influence of gender, differentially expressed genes based upon IGFBP2 concentrations (high/low) were analyzed with gender as a blocking factor. Three genes were identified as being significant, with lower expressions being associated with high IGFBP2 concentrations. Of these differentially expressed genes, 2 were identified as Y-linked genes (USP9Y and DDX3Y), while the third gene being Chromosome 4 open reading frame 3 (C4orf3) was not sex-linked (Fig 2a). Furthermore, results from hierarchical clustering showed that expression profiles did not form distinct clusters that were associated with circulating IGFBP2 concentrations (quartiles) (Fig 2b). Correlation analysis indicated that the primary tumor transcript levels for IGFBP2 did not correlate with circulating IGFBP2 concentrations in the matched samples (Fig 2c).

## Discussion

Relational biological databases can serve as powerful and reusable discovery tools for deepening the understanding of complex and interactive processes, such as cancer biology. Gleaning from collected scientific experiments, published investigative studies, and high-throughput experimental technologies, biological databases have the capacity to act as digital warehouses of information. These databases can be organized in varying combinatorial matrices to test preliminary hypotheses with the consequent acceleration of new exploratory questions that validate the merit of additional focused research.

**Fig 3:**
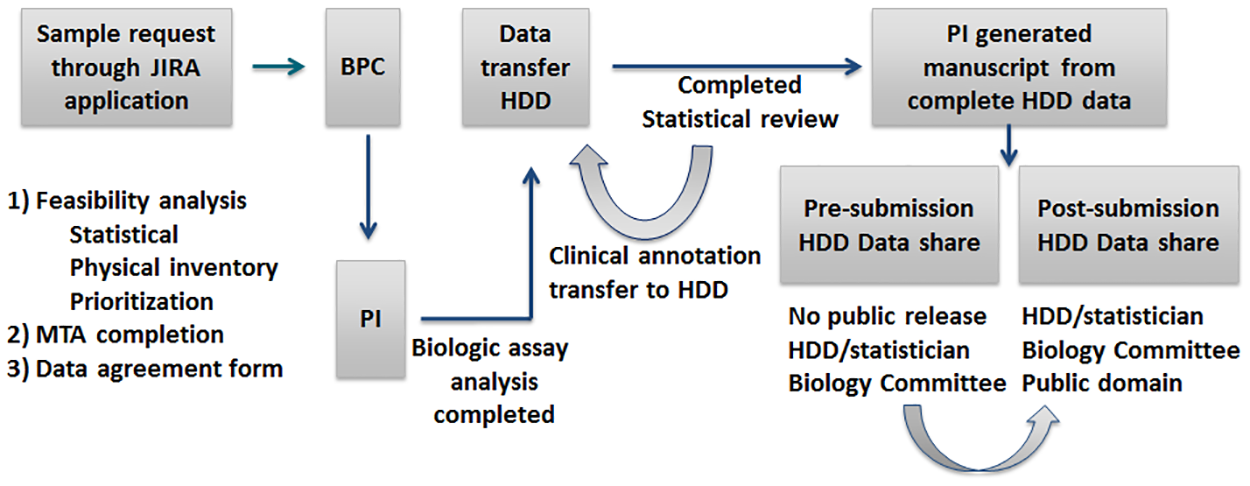
Proposed workflow for continued population of HDD with new digital datasets derived from voluntary sharing of research results provided by principal investigators utilizing exhaustible biospecimens.

The Children’s Oncology Group and QuadW Foundation have developed a relational biologic platform called the HDD to support the complexities of a wide diversity of data types collected within the work of a cooperative group. Through an agreed upon mechanism (Fig 3), the HDD will continue to evolve and expand through the proactive, yet voluntary, collection of investigator-derived data. Already through the voluntary sharing of generated results by osteosarcoma researchers, the HDD platform contains high-value mineable data from diverse biology projects including SPECS (gene microarray expression), TARGET (genome wide association studies), and serum proteomics. Additionally, datasets including germline TP53 variants and susceptibility to osteosarcoma, ezrin immunostaining, ErbB-2 status, telomerase expression, IGF-1/IGF-1R axis, chromosomal instability, and Fas expression allelotype/genotype are available for query. In efforts to streamline and support sarcoma biology research, the HDD platform will be accessible to independent investigators upon request to test hypotheses and quickly conduct *in silico* studies. In addition to expediting the generation of research questions, the HDD platform will also offer the potential to reduce the need for biospecimen requests and conserve the expenditure of finite physical specimen resources. The diverse data types collected in the HDD provide an unprecedented opportunity for researchers to ask formerly impossible questions because of sample availability or platform limitations. Equally important, the clinical and follow-up information of many biologic samples stored in HDD have been collected and available for *in silico* queries, which allows testing of translationally relevant hypotheses. Access to data digitally stored within the HDD platform will be made available to interested investigators following completion of standardized procedures that are analogous for biologic sample requests and might include statistical feasibility and scientific review.

To demonstrate the potential of the HDD platform to conduct *in silico* research, a proof-of-concept study was undertaken to associate unique datasets derived from 2 divergent investigations (pairs) which used overlapping patient-derived biospecimens. Specifically, the association of circulating IGFBP2 concentrations and primary osteosarcoma gene expression profiles based upon 46 patients with osteosarcoma were explored. Arising from this *in silico* research, when differential expression analysis was controlled for gender, the expression of 3 genes were identified to be significantly and inversely associated with circulating IGFBP2 concentrations, being USP9Y, DDX3Y, and C4orf3. Given the linkage of insulin-like growth factor-1 and associated binding proteins with gender [11], the identified inverse relationship of USP9Y and DDX3Y expressions, both Y-linked genes that play a role in spermatogenesis, with high IGFBP2 concentrations could be the residual effect of the imbalance gender distribution (13/20 being female) identified in the high IGFBP2 group. Nonetheless, both USP9Y and DDX3Y genes appear to participate in different forms of male reproductive tract cancers, suggesting they may play a role in tumorigenesis [12, 13]. The precise functions of these two Y-linked genes in osteosarcoma biology will need to be investigated experimentally, which is beyond the scope of this study. A novel but poorly annotated and non-sex linked gene, called C4orf3, was identified to have reduced expressions in primary osteosarcoma tumors where circulating IGFBP2 concentrations were categorized as high. The exact role of C4orf3 gene product remains unknown, however, this gene appears to be involved with some carcinoma subtypes (lung, head and neck) [14], and based upon its identification as a differentially expressed gene in osteosarcoma, additional research might be warranted to further elucidate its potential role in sarcomagenesis.

The results of unsupervised clustering showed that the distinct clusters formed by expression profiles of the primary tumors did not correlate with respective circulating IGFBP2 levels in the patients. These results suggest that concentrations of IGFBP2 alone are not directly linked with global gene expression similarities or differences among primary osteosarcoma samples. Given the known chaotic genomic landscape of osteosarcoma, perturbations in multiple signaling pathways are likely in sarcomagenesis, and the finding that IGFBP2 alone does not drive hierarchal clustering is not an unexpected scientific outcome. This result is also corroborated with the differential expression analysis, where only three genes were found significant; suggesting the influence of circulating IGFBP2 on tumor gene expression is not strong. Lastly, *in silico* analysis identified no association between measured circulating concentrations of IGFBP2 and primary tumor IGFBP2 mRNA transcription, indicating that other cellular sources in addition to osteosarcoma cells might contribute to total IGFBP2 concentrations. Additionally, other tumor and host factors including primary tumor size, histologic grade, and clinical stage of disease (macroscopic recurrence and/or metastases) that were not integrated into our *in silico* analysis could certainly confound our results and our reported findings should be interpreted with caution. As such, whether circulating IGFBP2 cannot be used as a direct biomarker of osteosarcoma burden requires additional investigation.

Although circulating IGFBP2 concentrations did not show a strong relationship with gene expressions profiles in the primary tumors within the current investigation, we have answered two important questions regarding the effect of circulating IGFBP2 on the primary tumors. We have demonstrated that the influence of IGFBP2 in the circulation have a minimal effect on the global gene expression in the primary tumors and only a small set of genes is differentially expressed between the high and low IGFBP2 groups. Also, the circulating IGFBP2 protein may not directly derived from the tumors. Hence, the ability to quickly test a hypothesis *in silico* was demonstrated through the use of the HDD platform. This case example serves to highlight the ease and feasibility of leveraging a relational database to answer biologically-driven questions. The continued population of the HDD platform with additional high-value and mineable datasets will increase the capacity to ask more sophisticated questions relevant to advancing sarcoma biology by both experimentalists and bioinformatic investigators alike. Ongoing efforts of the QuadW CSBAO and COG are to expand the HDD beyond osteosarcoma, and to include other childhood sarcomas including Ewing Sarcoma, Rhabdomyosarcoma, and Non-Rhabdomyosarcoma Soft Tissue Sarcomas. With increasing scientific community awareness and utilization of the HDD platform, it is expected that data sharing and transfer will become an accepted practice of independent researchers, allowing for tremendous potential benefits to the research community as a whole.

## Conclusions

The HDD platform serves as a relational database resource to facilitate discoveries relevant to sarcoma biology, and serves as an investigative resource available to the sarcoma research community. To utilize current datasets stored within the HDD platform, researchers should contact the QuadW-COG Childhood Sarcoma Biology and Annotation Office through datarequest@childrensoncologygroup.org.
